# Ethylene Glycol Intoxication Requiring ECMO Support

**DOI:** 10.1155/2021/5545351

**Published:** 2021-09-29

**Authors:** Raphael Rosen, Shelief Robbins-Juarez, Jacob Stevens

**Affiliations:** ^1^Department of Medicine, Division of Nephrology, Columbia University Irving Medical Center, 630 West 168th St, New York, NY 10032, USA; ^2^Columbia University Vagelos College of Physicians and Surgeons, 630 W 168th St, New York, NY 10032, USA

## Abstract

Ethylene glycol is commonly used in antifreeze, and ingestion of even a small amount can result in acute kidney injury, severe metabolic acidosis, and neurological injury. When cases are recognized early, treatment involves administration of alcohol dehydrogenase inhibitors to prevent conversion to toxic metabolites of glycolate, glyoxolate, and oxalate. In later presentations with more severe renal injury, hemodialysis may be required for clearance of toxic metabolites and supportive care for renal failure. We present the first reported case of severe ethylene glycol intoxication requiring support of extracorporeal membrane oxygenation (ECMO) due to refractory cardiopulmonary collapse.

## 1. Introduction

**Figure 1:**
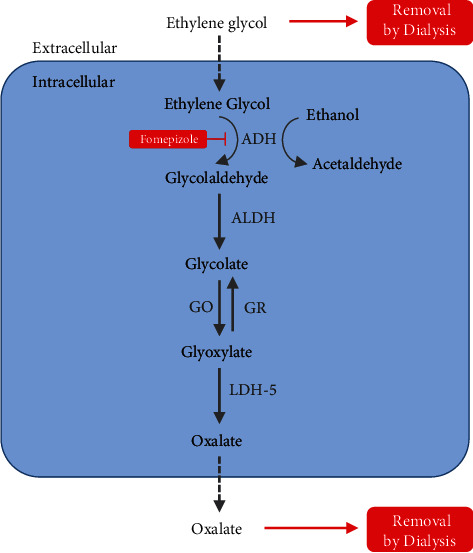
Ethylene glycol is metabolized via ADH to glycolaldehyde. Fomepizole and ethanol are competitive antagonists of ADH. The metabolic pathway leading from ethylene glycol to oxalate is shown. ADH: alcohol dehydrogenase; ALDH: aldehyde dehydrogenase; GO: glycolate oxidase; GR: glycolate reductase; LDH5: lactate dehydrogenase 5.

Ethylene glycol is present in several commonly available commercial products, notably automotive antifreeze, and cases of toxic ingestion have been reported as far back as the 1930s [1]. More recently, the 2019 Annual Report by the National Poison Data System (NPDS) recorded 7,260 cases of ethylene glycol intoxication, of which 874 were intentional, 2,287 received medical treatment, and 19 resulted in death [2]. Although ethylene glycol as a parent alcohol is relatively nontoxic, its metabolites—glycolate, glyoxylate, and oxalate—are nephrotoxic [3]. Oxalate inflicts damage via the formation of calcium oxalate crystals that cause tubular obstruction and hypocalcemia. This pathway is summarized in Figure 1. A peak serum concentration of 20 mg/dL is considered toxic and generally requires medical treatment, although case reports describe volumes as low as 20-30 mL, approximately one mouthful of antifreeze, to have caused somnolence and acute kidney injury (AKI) requiring dialysis [4]. In general, treatment focuses on alcohol dehydrogenase inhibition and supportive measures for AKI and acidosis, though in severe cases, hemodialysis may be required to facilitate clearance of both ethylene glycol and its metabolites. Rarely, cases may be severe enough to warrant more intensive supportive measures. Here, we report a dramatic case of ethylene glycol intoxication requiring the use of extracorporeal membrane oxygenation (ECMO).

## 2. Case Report

**Figure 2:**
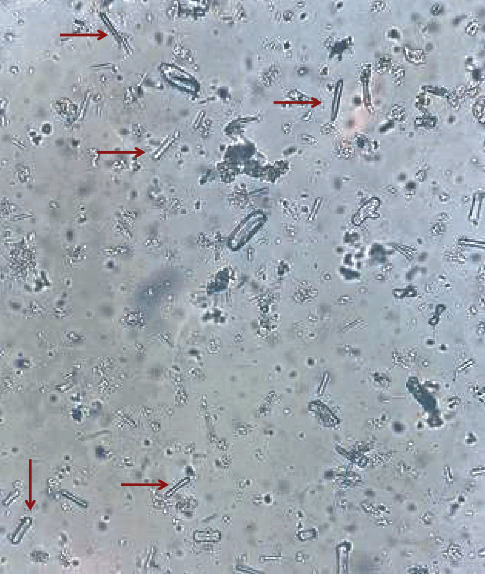
Arrows indicate calcium oxalate monohydrate crystals in this patient's urine sediment, viewed under nonpolarized light microscopy.

A 52-year-old woman with bipolar disorder with psychotic features and polysubstance use disorder was brought to the emergency department after being found at home with unsteady gait and altered mental status. Her partner reported that the patient had recently increased her alcohol consumption due to social and financial stressors. Upon the arrival of emergency medical services, the patient reported that she took 7 pills of bupropion and expressed suicidal ideation. The patient's prescribed medications were amlodipine, bupropion, and paliperidone. On initial examination, she was somnolent and barely rousable to physical stimuli. Noncontrast CT of the head ruled out any acute intracranial pathology. Initial laboratory values are detailed in Table 1, which were most remarkable for a severe metabolic acidosis with respiratory compensation and markedly elevated anion gap (the exact anion gap could not be calculated due to undetectable bicarbonate but was over 41). Serum beta-hydroxybutyric acid was normal, and urine ketones were negative. On a venous blood gas, the lactate was noted to be 3.5 mmol/L but venous lactate assay detected lactate of 29 mmol/L. Ionized calcium was low at 0.96 mmol/L. Serum osmolality was 320 mOsm/kg with a calculated osmolar gap of 16. Urine studies detailed in Table 2 were notable for a urine pH of 5 and an elevated oxalate to creatinine ratio. Calcium oxalate monohydrate crystals were present on microscopy (Figure 2). Urine drug and toxicology screens for acetaminophen, ethanol, salicylates, lithium, and valproic acid were all negative. Ethylene glycol, methanol, and metformin levels were sent.

Due to the high clinical suspicion for a toxic alcohol ingestion, and in consultation with poison control, the patient was volume resuscitated and given fomepizole, thiamine, and pyridoxine and was started on an intravenous bicarbonate infusion. She was admitted to the medical intensive care unit due to progressive somnolence. Repeat laboratory studies showed a rapidly rising Cr to 4 mg/dL and K to 6.7 mmol/L; her hyperkalemia was medically managed while a hemodialysis catheter was placed in anticipation of initiating dialysis. Immediately following dialysis catheter line placement but prior to initiation of hemodialysis, the patient developed bradycardia with widened QRS interval and then progressed to a sine wave tracing and loss of pulse. Advanced cardiac life support was initiated and return of spontaneous circulation was achieved after 45 minutes of CPR. She was subsequently cannulated to venoarterial extracorporeal membrane oxygenation (VA ECMO). After ECMO initiation, she was found to have cardiac standstill on echocardiogram and an Impella CP device was placed for left ventricular venting. She was started on continuous renal replacement therapy (CRRT) and underwent postarrest therapeutic hypothermia protocol. At this point, her confirmatory result for ethylene glycol returned positive at a level of 22 mg/dL; metformin and methanol were not detected. Her ethylene glycol level declined over the next two days (Table 3) and was undetectable by the third day of hospitalization, and fomepizole was stopped. She remained in severe shock with multiorgan failure complicated by shock liver, kidney injury requiring CRRT, respiratory failure requiring mechanical ventilation, rhabdomyolysis, and multiple episodes of asystole requiring inotropes and pressors. Her condition slowly stabilized and her cardiac function recovered to normal. She was decannulated from ECMO after 9 days of support. After transferring out of the ICU, she had persistent dialysis-dependent AKI and continued to have some residual neurological deficits including decreased strength and mobility, though her mental status returned to baseline. On subsequent questioning, she admitted to ingesting approximately 300 mL of antifreeze 8 hours prior to arriving to the ED. She consistently denied any further suicidal ideation and expressed regret over her suicide attempt, stating her motivation to continue to live for her family. She was discharged to a rehabilitation facility after an 85-day hospitalization.

## 3. Discussion

Signs and symptoms frequently associated with ethylene glycol intoxication include high anion gap metabolic acidosis, high osmolar gap, AKI, neurological dysfunction, and cardiopulmonary complications including cardiac arrest [5]. Treatment involves inhibition of alcohol dehydrogenase (ADH) using fomepizole or ethanol to block toxic metabolite formation and sodium bicarbonate to correct metabolic acidosis [6]. Pyridoxine can be used to shunt glyoxylate metabolism away from oxalate and toward less toxic metabolites [7]. Other novel therapeutics designed to inhibit enzymes in the pathway, notably lactate dehydrogenase 5, have recently been shown to have promising effects on urine oxalate levels and may play a role in future interventions [8].

On initial evaluation, the patient had many of the hallmark signs and symptoms of ethylene glycol intoxication, including lethargy, AKI with oxalate crystals, hypocalcemia despite marked acidosis, and severe anion gap metabolic acidosis with only mildly elevated lactate and normal serum ketones. In addition, there was a notable difference between lactate measured on venous blood gas (3.5) and venous lactate measured by laboratory assay (>29). This subtle finding of the so-called lactate gap is suggestive of ethylene glycol intoxication [9]. This is due to the structural similarity of glycolate and lactate, which interferes with certain assays used to measure lactate, such as the lactic oxidase method, but not the lactate dehydrogenase assay [10]. It is also important to note that the lack of a high osmolar gap is common in delayed presentations of toxic ingestion. The elevated serum osmolality occurs in the presence of the uncharged parent alcohol, which occurs early after ingestion. In delayed presentations, as in this case, most of the parent alcohol has been metabolized to monovalent anionic metabolites like glycolate, which are associated with cations like sodium and therefore do not contribute to the osmolar gap but do contribute to an anion gap [11]. Altogether, these findings in context of a history of substance abuse and suicidal ideation pointed strongly towards toxic alcohol intoxication.

The etiology of her refractory cardiac arrest was presumably a combination of severe acidosis and, considering her characteristic ECG changes, hyperkalemia. Notably, direct cardiotoxic effects of ethylene glycol leading to arrhythmia or cardiopulmonary collapse have also been described [12]. There have been few reported cases documenting the use of ECMO as a supportive measure for toxic ingestion and nothing available in the literature describing the use of ECMO specifically for ethylene glycol intoxication. Of the 26,271 cases of ethylene glycol intoxication reported to the American College of Medical Toxicology (ACMT) Toxicology Investigators Consortium (Tox-IC) Registry between 2010 and 2013, none of the 10 patients receiving ECMO were linked to ethylene glycol [13]. ECMO itself does not eliminate or neutralize toxins, but rather supports oxygenation, hemodynamics, and cardiac output while toxic metabolites are eliminated, either endogenously or extracorporeally via hemodialysis. Although there are no other available reports to compare outcomes, the utilization of ECMO in this case resulted in survival and recovery, suggesting that it can be useful in severe cases of cardiopulmonary collapse.

Although the patient demonstrated considerable clinical improvement, her long-term prognosis is unclear. In the largest study of long-term outcomes following ethylene glycol intoxication, of 5 patients who developed dialysis-dependent AKI, only one was dialysis-free after 18 months [14]. Most experienced continued neurological recovery with most rapid improvement occurring during the first year. In addition, there were 3 deaths from cardiopulmonary complications within 10 months of discharge. Given the patient's severity of illness resulting in a prolonged hospitalization and numerous complications, it is likely that she will have a prolonged path to recovery.

## 4. Conclusion

This case highlights a number of key diagnostic findings in ethylene glycol toxicity, including the presence of a severe anion gap metabolic acidosis, hypocalcemia, oxalate crystals in the urine, and the notable finding of discrepant lactic acid measurements on the blood gas analyzer and the venous lactate assay. It also serves as a reminder that the absence of an osmolar gap does not exclude toxic alcohol ingestion, particularly in delayed presentations. To our knowledge, this is the first report of ECMO being used to successfully support a patient with refractory cardiopulmonary collapse from ethylene glycol toxicity.

## Figures and Tables

**Table 1:** Serum lab tests.

BMP	Initial presentation	MICU presentation (9 hours after initial labs)
Sodium (137-145 mmol/L)	144	143
Potassium (3.5-5.1 mmol/L)	5.0	6.7
Chloride (98-107 mmol/L)	95	96
Carbon dioxide (19-27 mmol/L)	<7	<7
Blood urea nitrogen (7-26 mg/dL)	19	27
Creatinine (0.50-0.95 mg/dL)	2.20	4.00
Glucose (75-100 mg/dL)	160	231
Anion gap (5-17)	Not calculable	Not calculable
Calcium total (8.8-10.3 mg/dL)	10.0	8.6
Calcium ionized (1.12-1.32 mmol/L)	0.96	
Magnesium (1.6-2.6 mg/dL)	—	2.2
Phosphorous (2.5-4.5 mg/dL)	—	6.1
*ABG*		
pH (7.35-7.45)	7.05	7.11
pCO2 (32-45 mmHg)	11	10
pO2 (72-104 mmHg)	103	187
*CBC*		
WBCs (3.48-9.42 × 10^3^/*μ*L)	10.64	—
Hemoglobin (11.2-14.7 g/dL)	13.8	—
Platelet (167-374 × 10^3^/*μ*L)	287	—
*LFTs*		
Protein total (6.5-8.1 g/dL)	8.1	—
Albumin total (3.9-5.2 g/dL)	4.8	—
Bilirubin total (0.2-1.3 mg/dL)	0.2	—
Bilirubin direct (0.0-0.3 mg/dL)	0.1	—
AST (10-37 U/L)	18	—
ALT (9-50 U/L)	12	—
Alk Phos (40-129 U/L)	65	—
*Toxicology*		
Ethanol (mg/dL)	<10	—
Salicylate (<29.9 mg/dL)	<0.3	—
Acetaminophen (10-30 *μ*g/dL)	<5.0	—
Lithium (mmol/L)	<0.05	—
Valproic acid (*μ*g/dL)	<3.0	—
Urine drug screen	Negative	
*Other*		
Lactate (venous) (0.5-2.2 mmol/L)	>29	—
Lactate (blood gas analyzer) (0.5-2.2 mmol/L)	3.5	—
Creatinine kinase (40-308 U/L)	231.0	—
Serum osmolality (275-295 mOsm/kg)	320	—
Beta-hydroxybutyric acid (<0.27 mmol/L)	0.09	—

**Table 2:** Urine studies.

*Urinalysis*	
Color	Yellow
Appearance	Clear
Glucose	Negative
Bilirubin	Negative
Specific gravity (1.010-1.030)	1.016
Ketones	Negatives
Blood	Negative
pH	5.0
Protein	Trace
Urobilinogen (<2.0 mg/dL)	0.2
Nitrite	Negative
Leukocyte esterase	Negative
*Other*	
Urine osmolality	415
Urine calcium (mg/dL)	6.8
Urine uric acid (mg/dL)	<2.2
Urine oxalate (mg/L)	53
Urine creatinine (mg/dL)	68
Urine oxalate/Cr ratio (<32 mg/g)	78

**Table 3:** Ethylene glycol trend.

Time (hours after presentation)	7	14	27	31	48
Ethylene glycol (mg/dL)	22	20	15	14	<5
Anion gap (5-17)	>43	>41	28	25	11
Osmolar gap	16	—	—	—	—

## Data Availability

The underlying data were abstracted from the electronic medical record at Columbia University Medical Center/New York Presbyterian Hospital.
